# Early-stage differentiation between Alzheimer’s disease and frontotemporal lobe degeneration: Clinical, neuropsychology, and neuroimaging features

**DOI:** 10.3389/fnagi.2022.981451

**Published:** 2022-10-31

**Authors:** Pan Li, Wei Quan, Zengguang Wang, Ying Liu, Hao Cai, Yuan Chen, Yan Wang, Miao Zhang, Zhiyan Tian, Huihong Zhang, Yuying Zhou

**Affiliations:** ^1^Department of Neurology, Tianjin Huanhu Hospital, Tianjin, China; ^2^Department of Neurology, Tianjin Huanhu Hospital Affiliated to Tianjin Medical University, Tianjin Huanhu Hospital Affiliated to Nankai University, Tianjin University Huanhu Hospital, Tianjin, China; ^3^Tianjin Key Laboratory of Cerebral Vascular and Neurodegenerative Diseases, Tianjin Neurosurgery Institute, Tianjin Huanhu Hospital, Tianjin, China; ^4^Department of Neurosurgery, General Hospital of Tianjin Medical University, Tianjin, China; ^5^Tianjin Key Laboratory of Injuries, Variations and Regeneration of Nervous System, Tianjin Neurological Institute, Tianjin, China

**Keywords:** Alzheimer’s disease, frontotemporal lobar degeneration, differential diagnosis, neuropsychology, multimodal neuroimaging

## Abstract

**Background:**

Alzheimer’s disease (AD) and frontotemporal lobar degeneration (FTLD) are the two most common forms of neurodegenerative dementia. Although both of them have well-established diagnostic criteria, achieving early diagnosis remains challenging. Here, we aimed to make the differential diagnosis of AD and FTLD from clinical, neuropsychological, and neuroimaging features.

**Materials and methods:**

In this retrospective study, we selected 95 patients with PET-CT defined AD and 106 patients with PET-CT/biomarker-defined FTLD. We performed structured chart examination to collect clinical data and ascertain clinical features. A series of neuropsychological scales were used to assess the neuropsychological characteristics of patients. Automatic tissue segmentation of brain by Dr. Brain tool was used to collect multi-parameter volumetric measurements from different brain areas. All patients’ structural neuroimage data were analyzed to obtain brain structure and white matter hyperintensities (WMH) quantitative data.

**Results:**

The prevalence of vascular disease associated factors was higher in AD patients than that in FTLD group. 56.84% of patients with AD carried at least one APOE ε4 allele, which is much high than that in FTLD patients. The first symptoms of AD patients were mostly cognitive impairment rather than behavioral abnormalities. In contrast, behavioral abnormalities were the prominent early manifestations of FTLD, and few patients may be accompanied by memory impairment and motor symptoms. In direct comparison, patients with AD had slightly more posterior lesions and less frontal atrophy, whereas patients with FTLD had more frontotemporal atrophy and less posterior lesions. The WMH burden of AD was significantly higher, especially in cortical areas, while the WMH burden of FTLD was higher in periventricular areas.

**Conclusion:**

These results indicate that dynamic evaluation of cognitive function, behavioral and psychological symptoms, and multimodal neuroimaging are helpful for the early diagnosis and differentiation between AD and FTLD.

## Introduction

Alzheimer’s disease (AD) and frontotemporal lobar degeneration (FTLD) are two entities of major neurodegenerative disorders, leading to dementia, especially among young patients (<65 years old) (Neary et al., 1998; Dubois et al., 2014). Episodic memory impairment is usually the first symptom in the course of AD, however, variants of AD characterized by visual and language impairments have been well described and are termed as posterior cortical atrophy (PCA) (Benson et al., 1988; Crutch et al., 2012; Dubois et al., 2014) and logopenic variant primary progressive aphasia (lvPPA) (Mesulam, 2008; Gorno-Tempini et al., 2011; Dubois et al., 2014). A less common phenotype is the “frontal variant” of AD (fv-AD), with a clinical manifestations of mainly behavioral and/or executive disorders, which is easily misdiagnosed as FTLD (Dubois et al., 2014). Frontotemporal dementia (FTD) is a series of clinically heterogeneous disease, mainly manifested by behavioral abnormalities, language disorders, and executive function deficits. At present, it is classified into three major clinical types, including behavioral variant frontotemporal dementia (bvFTD) (Rascovsky et al., 2011), semantic variant of primary progressive aphasia (svPPA), and non-fluent variant primary progressive aphasia (nfvPPA) (Gorno-Tempini et al., 2011). Behavioral and executive disorders are predominant in bvFTD, while PPAs have severe language deficits. In addition, parkinsonism and motor neuron disease can be noted in many cases (Liu et al., 2019). No matter what symptoms appear, these disorders will develop overtime, and the symptoms will change with the course of the disease. Thereby, it may be difficult to establish an accurate diagnosis in the early stage of both diseases.

Since the clinical heterogeneity of the two disease spectrums, the early symptoms may be ambiguous and overlapping. The current clinical standard requires qualitative examination of clinical core symptoms and neuroimaging features, but due to the lack of high sensitivity and specificity, it is impossible to accurately differentiate AD from FTLD. Some clinical observational studies have shown that early episodic memory impairment should be the exclusion criteria for FTLD, but, actually, it is not absolutely (Katisko et al., 2019). Similarly, it is reported that behavioral and psychological symptoms are the characteristic manifestations of patients with FTLD, but some studies have shown that the proportion of neuropsychiatric symptoms in patients with AD can be as high as 93.4% (Laakso et al., 2000). Traditional visual assessment of brain MRI requires the time of an well-experienced neuroradiologist and provides only moderate sensitivity and specificity (Harper et al., 2016). Early diagnosis requires techniques, such as fluorodeoxyglucose-positron emission tomography (FDG-PET), to detect early brain changes, whereas its availability is limited and the costs is relatively high (Smailagic et al., 2015; Minoshima et al., 2021). When it is difficult to distinguish between AD and FTLD, the ways of computer-aided diagnosis may be useful. These methods utilize multivariate data analysis techniques to train models (classifiers) based on neuroimaging or related data, so as to realize objective diagnosis. Moreover, computer-aided diagnosis can take advantage of subtle between-group differences, which is more accurate than using only clinical criteria (Kloppel et al., 2012). Using structural MRI to discover characteristic patterns of brain atrophy, the accuracy of computer-aided diagnosis in the differentiation of AD and FTLD was yielded up to 84% (Raamana et al., 2014; Moller et al., 2016).

In addition to using structural MRI, evidence of neurodegeneration can also be surveyed by using advanced T2-weighted MRI sequences to detect the white matter hypersignal (WMH) changes, which have emerged as a potential biomarker of neurodegenerative diseases (Desmarais et al., 2021). Regional WMH is related to the clinical manifestation of AD and FTLD. In prospective longitudinal studies of elderly with normal cognition and AD patients, periventricular WMH was negatively correlated with mental processing speed, and WMH in left temporal lobe was negatively correlated with memory performance (Smith et al., 2011; Ramirez et al., 2014). However, the neural correlates of WMH have not been extensively and rigorously studied in AD and FTLD. Mapping the distribution and burden of WMH in AD and FTLD can provide further insight into the underlying pathological mechanisms.

Although both diseases and their subtypes have been well incorporated into new diagnostic criteria (Gorno-Tempini et al., 2011; Rascovsky et al., 2011; Dubois et al., 2014), little is known about the initial symptoms, risk factors, genetic susceptibility, behavioral and neuropsychological characteristics and common pathological characteristics of these phenotype. It is necessary for better understanding of neurodegenerative diseases across the boundaries of different clinical entities, as it likely improves the ability of clinicians to identify the histopathological cause of dementia. We enrolled a large number of patients with AD or FTLD defined by biomarkers or neuroimaging in this retrospective study. In the present study, we aimed to better represent the clinical, neuropsychological, and neuroimaging features. We attempted to present a framework that contains a series of volume measurements of different brain tissues to supply clinical information for differential diagnosis of AD and FTLD. We also investigated the burden and distribution of WMH in these neurodegenerative diseases and studied the correlation of neuropsychiatric manifestations with brain WMH.

## Materials and methods

### Subjects and inclusion criteria

Two hundred one subjects were screened and included in the study, and a case-control clinical-imaging observational prospective study was conducted by the Dementia Research Institute (DRG) at Tianjin Huanhu Hospital between 2012 and 2021. All enrolled patients completed a standardized research battery of validated tests and multisequence imaging by a 3.0-T MRI scanner (MAGNETOM ESSENZA, Siemens Healthineers, Germany and Signa HDxt, GE Healthcare, USA) and partially by 18-fluorodeoxyglucose-positron emission computed tomography (^18^FDG-PET-CT) and Pittsburgh compound B-positron emission computed tomography (^11^C-PET-CT). All of them were assessed by at least 2 experienced specialists in the field of dementia. Patients who were clinically diagnosed with typical AD fulfilled the criteria for probable AD dementia as defined by the National Institute on Aging-Alzheimer’s Association (NIA-AA) (McKhann et al., 2011) and the variants of AD fitted the International Working Group (IWG) –2 criteria (Dubois et al., 2014). FTLD patients met the clinical criteria for the FTLD disease spectrum (Gorno-Tempini et al., 2011; Rascovsky et al., 2011). Exclusion criteria: (1) those with disturbance of consciousness, severe aphasia or serious illness, unable to complete the evaluation of neuropsychological scale; (2) symptoms caused by other systemic diseases or non-degenerative diseases of nervous system; (3) patients with heart, lung, liver, kidney, endocrine system diseases, or serious medical diseases such as connective tissue disease, hematopathy, and malnutrition; (4) patients with a history of brain or other tumors, brain trauma, gas poisoning, long-term alcoholism, epilepsy, etc.; (5) abnormal behavior conforms to psychiatric diagnosis; (6) biomarkers indicate other neurodegenerative diseases (non-AD or FTLD).

### Ethical considerations

All the subjects were accompanied by reliable caregivers, and the subjects and their families signed the informed consent form. All procedures are carried out according to the ethical standards specified by Tianjin Human trial Committee and approved by Ethics Committee of Tianjin Huanhu Hospital.

### Clinical evaluation and procedures

Baseline demographics and clinical data were collected through comprehensive geriatric assessment to ensure confidentiality. Participants underwent a series of detailed neurological examinations to evaluate their clinic symptomatic status, cognitive and both behavioral and neuropsychiatric performances at baseline by the experienced, board-certified neurologist. The Mini-Mental State Examination (MMSE) (Molloy and Standish, 1997) and Montreal cognitive assessment scale (MoCA) (Hu et al., 2013) were applied to assess subjects’ cognitive function, and the scores of each sub-item were recorded in detail. The Clinical Dementia Rating (CDR) (Morris, 1993) was used to estimate the grade of dementia. Behavioral and psychological symptoms evaluation: Neuropsychiatric Inventory Questionnaire (NPI) (Wang et al., 2012) and Frontal Behavioral Inventory (FBI) (Kertesz et al., 2000) were applied to evaluate the psychobehavioral symptoms of the subjects. Daily activity ability and emotional state of patients were evaluated by Activities of Daily Living (ADL) Scale (Eto et al., 1992) and Hamilton Depression scale 21 (HAMD-21), respectively (Faries et al., 2000).

### Neuroimaging and biochemical assessment

Positron emission tomography (PET) and CSF estimation of each diagnostic group were shown in Table 1. ^18^F-FDG-PET-CT was performed to assess patterns of hypometabolism across the brain. Amyloid PET using ^11^C-Pittsburgh compound B (PiB) (Klunk et al., 2004) and/or amyloid-β_42/40_ biomarkers in CSF were applied for pathological evaluation.

**TABLE 1:** Baseline demographics and clinical characteristics of AD and FTLD groups.

Variables	AD	FTLD	Statistic value	*P*-value
Enrolled patients (n)	95	106	—	—
Age (mean ± SE, yrs.)	68.23 ± 0.95	63.24 ± 0.80	7.295 ^b^	0.000*
Gender (M/F)	51/44	45/61	2.533 ^a^	0.111
Course of disease (mean ± SE yrs.)	3.05 ± 0.31	2.03 ± 0.21	2.344 ^b^	0.021*
Age of onset (mean ± SE, yrs.)	64.84 ± 1.17	59.94 ± 0.94	3.239 ^b^	0.002*
**Marital status (n [%])**				
Married	95	106		
Not married	0	0	2.425 ^a^	0.297
Widow (er)	7	15		
**Dwelling state**				
Living with Family	86	101		
Solitary	9	5	1.749 ^a^	0.186
BMI (Mean ± SE, kg/m^2^)	22.89 ± 0.45	23.89 ± 0.36	–1.744 ^b^	0.083
Education (Mean ± SE, yrs.)	10.68 ± 0.42	9.93 ± 0.41	1.204 ^b^	0.230
Smoking (n [%])	29 (30.52)	13 (12.26)	10.108 ^a^	0.001*
Drinking (n [%])	23 (24.21)	9 (8.49)	9.249 ^a^	0.002*
Dementia family history (n [%])	13 (13.68)	29 (27.36)	70.426 ^a^	0.000*
**Vascular diseases**				
Hypertension (n [%])	35 (36.84)	28 (26.41)	2.531 ^a^	0.112
Heart disease (n [%])	20 (21.05)	15 (14.16)	1.659 ^a^	0.198
Diabetes (n [%])	24 (25.26)	15 (14.15)	3.956 ^a^	0.047 *
Hyperlipemia (n [%])	14 (14.74)	6 (5.66)	4.606 ^a^	0.032 *
Stroke history (n [%])	5 (5.26)	6 (5.66)	0.015 ^a^	0.902
TC (Mean ± SE, mmol/l)	5.85 ± 0.25	5.29 ± 0.16	1.773 ^b^	0.080
LDL-C (Mean ± SE, mmol/l)	3.43 ± 0.11	2.77 ± 0.20	1.553 ^b^	0.125
TG (Mean ± SE, mmol/l)	1.37 ± 0.07	1.30 ± 0.11	0.542 ^b^	0.590
HDL-C (Mean ± SE, mmol/l)	1.45 ± 0.05	1.39 ± 0.06	0.807 ^b^	0.422
APOE ε4 carriers (n [%])	54 (56.84)	16 (15.09)	38.470 ^a^	0.000*
AD7c-NTP (Mean ± SE, ng/ml)	3.36 ± 0.24	2.82 ± 0.35	1.283 ^b^	0.205
^18^FDG-PET-CT/^11^C-PET-CT/CSF Aβ_42/40_ biomarker (n)	95/11/44	82/9/26	—	—
Structural MRI	35	54	—	—
MMSE	17.47 ± 0.63	18.91 ± 0.60	–1.639 ^b^	0.103
CDR	1.39 ± 0.11	1.36 ± 0.15	0.187 ^b^	0.852
MoCA	18.64 ± 0.74	15.56 ± 1.17	2.348 ^b^	0.022 *
NPI	8.93 ± 0.87	15.75 ± 2.51	–3.15 ^b^	0.002 *
FBI	16.83 ± 1.44	24.05 ± 2.45	–2.649 ^b^	0.010 *
FBI-A	9.15 ± 0.81	13.30 ± 1.36	–2.734 ^b^	0.008 *
FBI-B	7.94 ± 0.76	10.75 ± 1.19	–2.004 ^b^	0.049 *
ADL	32.27 ± 1.41	37.21 ± 1.65	–2.116 ^b^	0.036
BADL	13.43 ± 0.65	14.84 ± 0.69	–1.417 ^b^	*0.159
IADL	18.37 ± 0.87	21.91 ± 0.99	–2.536 ^b^	0.012 *
HADM-21	6.51 ± 0.56	9.21 ± 0.80	–2.738 ^b^	0.007 *

^a^ is χ^2^ statistic value and analyzed with Chi-square test, ^b^ is *t* statistic value and analyzed with independent sample *t*-tests; * *P* < 0.05 vs. FTLD group.

AD, Alzheimer’s disease; AD7c-NTP, Alzheimers disease associated neural filament protein; ADL, Activity of Daily Life; BADL, Basic Activity of Daily Life; IADL, Instrumental Activity of Daily Life; APOE, Apolipoprotein E; BMI, Body Mass Index; CDR, Clinical Dementia Rating Scale; F, female; FBI, Frontal Behavioral Inventory; FBI-A, Frontal Behavioral Inventory, Positive term subscale; FBI-B, Frontal Behavioral Inventory, Negative term subscale; FTLD, Frontotemporal Lobar Degeneration; HADM-21, The 21-items Hamilton Depression Rating Scale; HDL-C, High density Lipoprotein Cholesterol; LDL-C, Low density Lipoprotein Cholesterol; M, male; MMSE, Mini-Mental State Examination; MoCA, Montreal Cognitive Assessment; NPI, Neuropsychiatric Inventory Questionnaire; PET, Positron Emission Computed Tomography; TC, Total Cholesterol; TG, Triglyceride.

On the second day after admission, morning blood or serum specimen was gathered after fasting at night. The levels of total cholesterol (TC), triglyceride (TG), high-density lipoprotein cholesterol (HDL-C), and low-density lipoprotein cholesterol (LDL-C) were gauged by ADVIA 2400 automatic biochemical analyzer (Siemens, Germany). The urine of AD-associated neural filament protein (AD7c-NTP) was also assessed.

### Magnetic resonance imaging gathering and processing

Structural MRI scans were collected in our study: (1) T1-weighted MR images were obtained by a 3D magnetization-prepared rapid spin-echo (MPRAGE) sequence: repetition time/echo time (TR/TE) = 2530/3.43 ms; FA = 90°, matrix size = 256 × 256; field of view (FOV) = 265 × 224 mm^2^; slice thickness = 1.0 mm; gap = 1.2 mm; (2) T2 fluid attenuated inversion recovery (T2 FLAIR) image: TR/TE = 8000/120 ms; matrix size = 512 × 512; field of view (FOV) = 240 × 240 mm^2^; slice thickness = 1.2 mm; gap = 1.2 mm. The proposed method was preceded following the previously published description (Jiang et al., 2020; Wei et al., 2020). First, skull dissection was performed on T1W and T2 FLAIR images using FMRIB software library.^1^ Then, based on rigid transformation and normalized mutual information, the T2 FLAIR images of skull dissection were aligned and registered to T1WI images through SPM12 (Ashburner and Friston, 2011). N4 deviation correction was then operated on T1W and T2 FLAIR images to eliminate low-frequency intensity heterogeneity.^2^

Brain structure and WMH quantitative data were analyzed using Dr. Brain analysis system (^3^registration number: 20212210359), which has its own standard healthy population database platform as a control. All patients’ T1WI and T2 FLAIR DICOM data were compressed into Zip files and simultaneously uploaded to Dr. Brain cloud system for image analysis. This system is an automatic segmentation based on multi-template segmentation. A patient image is processed in the Dr. Brain cloud system for approximately 25 min to automatically generate a PDF report containing the information of absolute and relative (that is, the percentage of absolute volume of WMH in the total intracranial volume) volume of WMH of each brain region and brain regional volume (the total volume, parenchyma and brain white matter of more than 100 brain regions were recorded). Statistical parameter mapping (SPM)-voxel-based morphological analysis (VBM) and surface-based morphometry (SBM) were applied to quantify changes in gray matter structure under pathophysiological conditions. The differences of gray matter density (GMD) between groups were analyzed and implemented by VBM8 toolbox^4^ and SPM8 (Wellcome Trust Centre for Neuroimaging, London, UK). Data were pre-treated on the basis of VBM8 toolbox. The results were controlled by potential confounding factors, including age, gender, total intracranial volume, and MRI equipment. For family-wise error on the cluster level, *P* value less than 0.01 was set as analysis threshold.

We observed four parameters in SBM: cortical thickness, sulcus depth, gyrification index, and fractal dimension of cortical complexity. The cerebral cortex is a highly folded sheet of gray matter (GM), with areas that fold inward called sulci and areas that fold outward called gyri. There are three commonly used surfaces to describe this sheet: outer surface, inner surface, and central surface (CS). Cortical thickness describes the distance between the inner and outer surfaces (Dahnke et al., 2013). Sulcal depth computation will be processed according to the following procedures (Lyu et al., 2018): first, interiors of the cortical surface are filled in the volumetric space. Then, the cerebral hull is obtained by closing sulci through a three-dimensional spherical morphological closing operation. Next, the volumetric Boolean operation defines the intersection between the exterior of the cortical surface and the interior of the cerebral hull, which is severed as a medium between those two interfaces. Finally, the geodesic distance is calculated inside the medium by solving an Eikonal equation. The actual depth calculation (wavefront propagation) is performed on multiple slices of the volume. The gyrification index is extracted from central surface data, based on absolute mean curvature, which is the mean curvature calculated from the average between the minimum and maximum curvatures of the surface in each vertex in mm^–1^ – the mean curvature maps will, hereafter, referred to as the gyrification index (Chaudhary et al., 2021). Fractal dimension is a quantitative indicator of the morphological complexity and variability of an object. The different metrics for measuring fractal dimension are Hausdoff dimension, box counting dimension, capacity dimension, and mass radius dimension. There is increasing evidence that shape analysis using fractal dimension provides better information about structural changes induced by neurological conditions, which can supplement the information obtained by conventional volumetric analysis (Sheelakumari et al., 2018).

### Positron emission tomography image acquisition and processing

The acquisition and processing protocols for 18F-FDG and 11C-PIB PET imaging have been described in our previous study (Zhang et al., 2017; Wang et al., 2019). Briefly, PET images were acquired in the three-dimensional scanning mode on a GE Discovery LS PET/CT 710 scanner. 11C-PIB was administered intravenously at a dose of 370-555MBq, and a 90-min dynamic PET scan was performed according to a predetermined protocol. One hour after the 11C-PiB PET scan, 185-259 MBq of 18F-FDG was then injected intravenously, A 10-min static PET emission scan was performed 40 min after FDG injection with the same scanning mode. FDG PET and PiB PET images were preprocessed using MRI data for partial volume effect correction and spatial normalization. PiB PET imaging analysis was performed using Statistical Parameter Mapping 8 (SPM8) software on MATLAB 2010b for Windows (Mathworks, Natick, MA, USA) or PMOD software (version 3.7, PMOD Technologies Ltd., Zurich, Switzerland), as described in our previous study. The average of all specific regions was calculated from the PiB integral image. FDG frames for each subject were summed and normalized to mean pons activity. It is then displayed on the NIH color scale and can be windowed and viewed on three planes according to the rater’s discretion.

### Visual rating for Pittsburgh compound B positron emission tomography and fluorodeoxyglucose-positron emission tomography

The PiB PET and FDG PET imaging results were evaluated by two experienced nuclear medicine physicians. The positivity or negativity of PiB PET was determined by the mean value of target regions to cerebellum ratio with a cutoff value of 1.5 (the upper 95% confidence interval from a cluster analysis of healthy individuals). 18F-FDG PET images were read with color scale and standard to preferably route clinical brain FDG PET reports. FDG PET images were graded and dichotomized as follows: temporoparietal cortex dominant hypometabolism, other brain regions dominant hypometabolism or non-specific and mild hypometabolism.

### Statistical analysis

Chi-square test was used to examine the baseline demographic qualitative variables, described as the relative abundance ratio (%) or rate (%). Normally distributed quantitative variables were calculated using two independent sample *t*-tests or one-way analysis of variance (ANOVA). All values were presented as mean ± standard (SD) deviation. All statistical analyses were performed with SPSS 26.0 (SPSS, Inc., USA). A P value <0.05 was considered statistically significant.

### Data availability

Anonymized data can be obtained from the corresponding author upon request from any qualified researcher to replicate protocols and results.

## Results

### Demographic, biomarker measurements, and neuroimaging of the study population

Demographic, clinical characteristic data, and biomarker measurements were summarized in Table 1. We recruited 95 AD patients (51 men and 44 women) and 106 FTLD patients (45 men and 61 women). All the diagnoses of the participants were confirmed by ^18^FDG-PET-CT or ^11^C-PET-CT combined with cerebrospinal fluid amyloid-β 42 ratio 40 (Aβ_42/40_) biomarker results. Patients with FTLD were relatively young at time of disease onset and diagnosis (mean age: age at onset, 59.94 ± 0.94 years; age at diagnosis, 63.24 ± 0.80 years) and had more shorter time to diagnosis (mean time, 2.03 ± 0.21 years) compared with AD patients (mean age: age at onset, 64.84 ± 1.17 years; age at diagnosis, 68.23 ± 0.9 years; course of disease, 3.05 ± 0.31). Smoking, drinking, and risk factors associated with vascular disease (diabetes, hyperlipemia) were more frequent in AD patients; however, the genetic predisposition in FTLD families is more pronounced than in patients with AD. Apolipoprotein E (APOE) ε4 allele was more common in the AD group. APOE ε4 alleles occurred in 56.84% of patients in the AD group and 15.09% of patients in the FTLD group.

Cognitive function analysis showed that there was no remarkable difference in the total score of MMSE and CDR between the two groups, but the MoCA scores of FTLD patients were lower than that of AD patients. FTLD patients had more severe psychobehavioral symptoms, mood disorders, and executive dysfunction as compared with AD patients. Although not statistically significant, patients with FTLD tended to decrease in the ability to perform daily activities and had higher scores on the instrumental activity of daily life (IADL).

**FIGURE 1:**
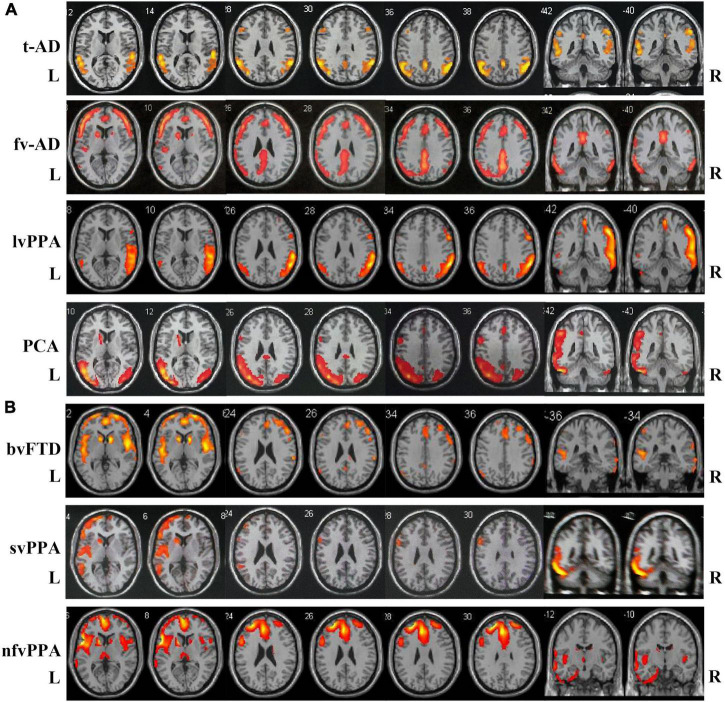
Atrophy maps in different clinical subtypes of AD and FTLD disease groups analyzed by ^18^FDG-PET-CT. **(A)** Typical AD is characterized by predominant temporoparietal cortex atrophy, fv-AD will partially spread to frontal cortical regions. LvPPA displayed predominant posterior perisylvian or parietal atrophy. PCA mainly involved temporo-occipital cortex atrophy. **(B)** In FTLD disease group, bv-FTD showed frontal and anterior temporal atrophy, svPPA was more frequent with involvement of anterior temporal, and nfvPPA patient showed more posterior fronto-insular atrophy. t-AD, typical Alzheimer’s disease; fv-AD, frontal variant Alzheimer’s disease; lvPPA, logopenic variant primary progressive aphasia; PCA, posterior cortical atrophy; bv-FTD, behavioral variant frontotemporal dementia; svPPA, semantic variant of primary progressive aphasia; nfvPPA, non-fluent variant primary progressive aphasia.

Typical neuroimaging features of ^18^FDG-PET-CT in different subtypes of two disease groups are summarized in Figure 1. Typical AD patients exhibit a typical default network pattern, with hypometabolic effects in the temporoparietal junction areas including precuneus and posterior cingulate cortex. Frontal variant in AD group was more frequently involved in the frontal cortical areas (such as anterior cingulate, orbitofrontal cortex, middle and superior frontal gyrus) compared with typical AD patients. Logopenic variant of AD displayed predominant posterior perisylvian or parietal hypometabolism. In FTLD groups, behavioral variant FTD patients showed frontal and anterior temporal hypometabolism on PET, svPPA patients showed more frequently relative involvement in the anterior temporal, and nfvPPA patients showed more involvement in the posterior fronto-insular.

### Clinical presentations comparison among diagnostic groups

**FIGURE 2:**
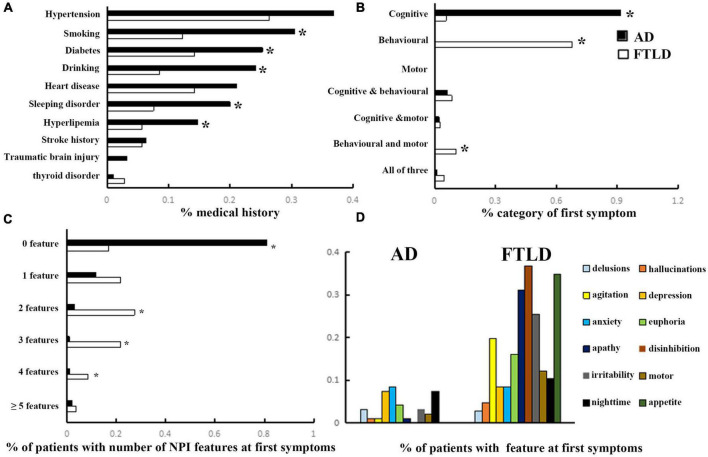
Clinical features comparison between AD and FTLD disease groups. **(A)** Past medical conditions in self-reported or supplied by caregivers. **(B)** Rate of first symptoms informed by patients and caregivers. **(C)** Frequency of psychobehavioral abnormalities in the first episode. **(D)** Clinical features of psychobehavioral abnormalities in the first symptom.

In the study population, hypertension, diabetes, heart disease, sleeping disorder, hyperlipemia, stroke history, and traumatic cerebral injury were the most frequently noted diseases in the history of medicine. The incidences of diabetes and hyperlipemia histories in AD were remarkably higher than that in FTLD (confounding factors were corrected by logisitc regression analysis). The proportions of AD patients with smoking and drinking habits were also higher than those with FTLD patients. However, the incidence of thyroid disease is higher in FTLD than in AD (Figure 2A).

The frequency of cognitive impairment (91.6%) was higher than that of behavioral changes (6.3%) in AD patients. Conversely, patients with FTLD predominantly presented with behavioral deficits (67.9%) or both behavioral and motor dysfunction (10.4%) as the first symptoms of the disease (Figure 2B). Although all of the FTLD patients were satisfied, the diagnostic criteria of ≥3 of 6 core behavioral/cognitive symptoms required to diagnose possible behavioral variability FTD at the time of diagnosis, most patients were only near the threshold value of diagnostic criteria at the onset of symptoms [0 feature: 18/106 (16.98%), 1 feature: 23/106 (21.70%) or 2 features: 29/106 (27.35%), (Figure 2C)], and had more behavioral symptoms compared with AD patients. Among the neuropsychiatric list fields frequently mentioned in FTLD population, apathy, disinhibition, and appetite were the most prominent, followed by agitation and irritability, which were less common (Figure 2D).

### Changes in neuropsychological and neuropsychiatric symptoms among diagnostic groups

**FIGURE 3:**
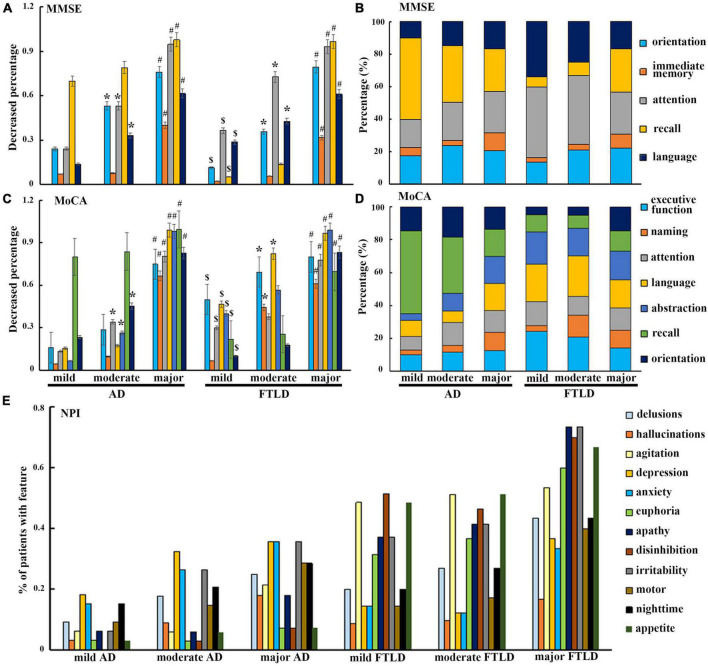
Neuropsychological characteristics comparison between AD and FTLD disease groups with disease progression. **(A–D)** Decreased degree of subscale items in MMSE and MoCA assessment with disease progression. **(E)** Frequency of neuropsychiatric symptoms in different stages of disease measured by NPI.

In order to avoid the influence of the severity of dementia, patients in AD group and FTLD group were divided into three grades: CDR1 stage (AD 33 cases, FTLD 27 cases), CDR2 stage (AD 45 cases, FTLD 57 cases), and CDR3 stage (AD 17 cases, FTLD 22 cases). In the evaluation of cognitive function, AD patients mainly showed decreased delayed recall ability in the early stage, problems in attention, orientation, executive function, and language became more and more obvious as the disease progressed (Figures 3A–D). However, in FTLD patient group, attention, executive and language dysfunction, and decreased abstraction were always the earliest symptoms. In the late stage, overall cognitive decline and memory impairment were observed (Figures 3A–D). In neuropsychiatric behavior evaluation, patient group with FTLD showed worse neuropsychiatric functions than AD group, almost in all subdomains. Patients with AD generally developed behavioral abnormalities in the middle and later stages of the disease, mainly manifested as depression, anxiety, and irritability. In patients with mild-to-moderate FTLD, agitation, disinhibition, and appetite were the first prominent behavioral symptoms, then followed by euphoria, apathy, and irritability, and finally developed into a comprehensive spectrum involved. Hallucination was the least common symptom, even in the advanced stages of FTLD (Figure 3E).

### Volumetric feature differences of brain regions among diagnostic groups

**FIGURE 4:**
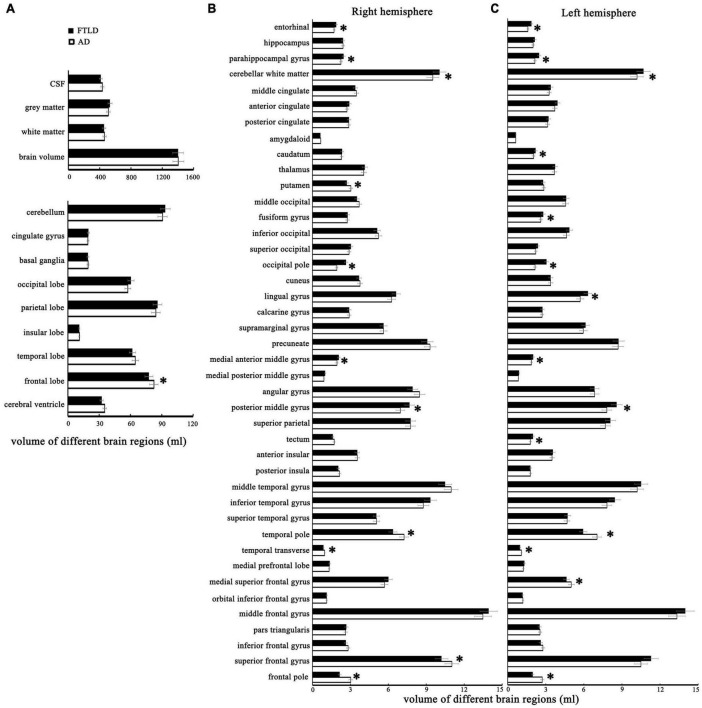
The structural MRI features with statistical differences between patients with AD and FTLD. **(A)** Comparison of different brain structures between the two groups. **(B)** Comparison of the cortical volume of different brain regions in the right cerebral hemisphere between the two groups. **(C)** Comparison of the cortical volume of different brain regions in the left cerebral hemisphere between the two groups.

Volumetric differences between AD and FTLD, as well as the differences between each group with healthy controls, are summarized in Figure 4. The structural changes of brain regions in patients with AD and FTLD were obvious. Compared with FLTD, AD patients have a larger frontal lobe volume and a tendency to have larger superior frontal gyrus and frontal pole volume in their subdomains. Although there were no significant difference in the overall volume of temporal lobe, occipital lobe, parietal lobe, basal ganglia, and cerebellum between the two groups, subfields analysis showed that the volumes of temporal polar and transtemporal region were more preserved in AD patients, and the volumes of parietal, occipital lobe and cerebellum subareas were relatively less. There was no difference in hippocampal volume between the two groups, however, bilateral parahippocampal gyrus and entorhinal cortex volumes decreased in AD patients. In FTLD individuals, there is asymmetric atrophy of the right putamen and left caudate nuclei in the subcortical basal ganglia region.

**FIGURE 5:**
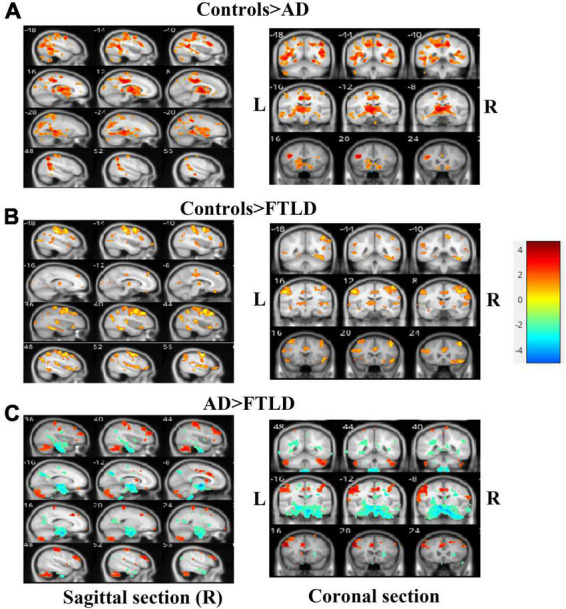
Voxel-based morphometry comparisons of gray matter volumes in AD or FTLD patients compared with healthy controls. The contrasts were adjusted according to age, gender, total intracranial volume and scanner type. **(A)** Comparison between AD patients and healthy controls; **(B)** Comparison between FTLD patients and healthy controls; **(C)** FTLD patients compared with AD patients. Color overlay shows *p*_unc_ < 0.001 for family-wise error = 0.05. The warm color of the color bar representing gray matter volume of controls lager than AD or FTLD, and cold color denoting gray matter volume of controls smaller than AD or FTLD **(A, B)**. The warm color of the color bar representing gray matter volume of AD lager than FTLD, and cold color denoting gray matter volume of AD smaller than FTLD **(C)**.

We performed VBM comparisons between the different patients and control groups (Figure 5), and trimmed for age, gender, disease severity, and total intracranial volume. Compared with the healthy control group, the characteristic pattern of brain atrophy in AD patients involved a large area of the temporoparietal cortex, posterior cingulate gyrus, precuneus, and part of the occipital lobes. The frontal cortex is also partially involved in AD patients (Figure 5A). In FTLD group, the affected atrophy areas were mainly concentrated in the frontal pole, orbito-frontal lobe, frontal insula, anterior cingulate gyrus, and bilateral anterior temporal lobes comparing to healthy control group. It also affects the parietal part of the posterior central gyrus (Figure 5B). Direct comparison between patient groups showed the posterior involvement of AD patients and the anterior involvement in FTLD patients had significantly different atrophy patterns, which mainly survived the correction of family-wise error (FWE) with *P* > 0.05. FTLD patients showed asymmetry in the affected brain regions (Figure 5C).

**FIGURE 6:**
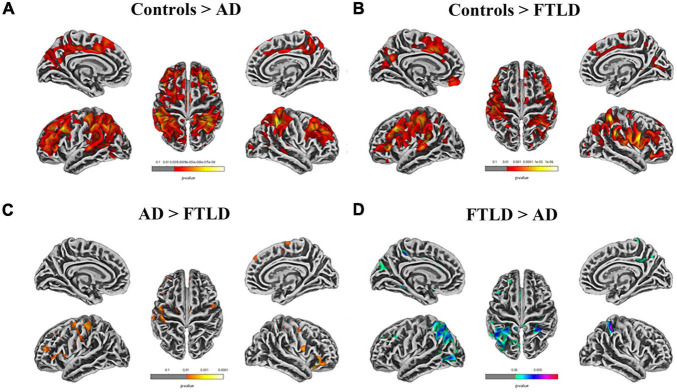
Cortical thickness alterations in different groups assessed by surface-based morphometry. **(A)** Comparison between AD patients and healthy controls; **(B)** Comparison between FTLD patients and healthy controls; **(C, D)** FTLD patients compared with AD patients. Color overlay shows *p*_unc_ < 0.001 for family-wise error = 0.05.

In the SBM-based structural brain characterization, differences in cortical thickness (Figure 6), sulcus depth, gyrification index, and fractal dimension (Supplementary Figure 1) between the two disease groups of patients and healthy controls were analyzed. The results showed that there were disease-specific alterations in brain structure in both AD and FTLD groups compared with the control group (Figures 6A,B). In the AD group, cortical thickness in the superior parietal lobe, inferior parietal, superior temporal, lateral occipital, fusiform, and rostral middle frontal lobe decreased symmetrically (Figure 6A). However, in FTLD patient group, the cortical thickness of the supramarginal, caudal middle frontal, pars triangularis, superior frontal, and superior temporal decreased significantly, and the distribution tended to be asymmetrical (Figure 6B). As compared to AD patients, the FTLD patient group showed a reduction in pars triangularis, rostral middle frontal, lateral orbitofrontal (Figure 6C), whereas AD exhibited a significant reduction in the superior parietal, lateral occipital, precuneus cortex as compared to FTLD patient group (Figure 6D) (voxel significance set to *P* < 0.01 or 0.001, corrected significance set to *P* < 0.01). The AD and FTLD patient groups exhibited characteristic changes in sulcus depth, gyrification index, and fractal dimension (Supplementary Figure 1) between the AD and FTLD patient groups as compared to the healthy controls and intercomparison. Specific brain areas involved are shown in the Supplementary Table 1.

### White matter hyperintensities volumetrics and characteristics among diagnostic groups

**FIGURE 7:**
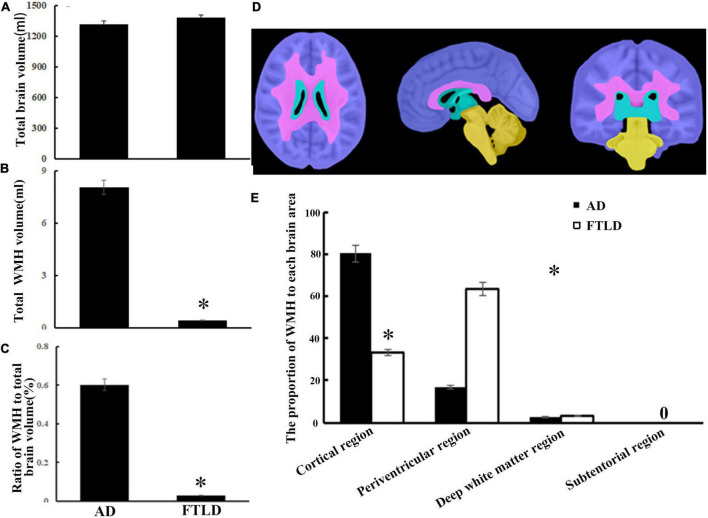
White matter hyperintensity volumes and distribution comparison between AD and FTLD disease groups. **(A)** Total brain volume comparison. **(B)** Total WMH volume comparison. **(C)** Total WMH ratio comparison. **(D)** Diagram of white matter region division supplied by semi-automatic brain region extraction. **(E)** Comparison of the proportion of WMH in different brain regions.

According to different brain regions, white matter signals can be divided into cortical, periventricular, deep white matter, and subatentorial regions. There was no significant difference in total cerebral capacity between the two groups after checking for blood vascular hazard factors and age at the time of imaging (Figure 7A), but there were significant differences in WMH load and brain distribution between the study groups (Figures 7B–E). There were significant differences between groups in the total burden of WMH, and the average volume of AD group (8.05 ± 3.25 ml) was higher than that of FTLD group (0.60 ± 0.030 ml) (Figures 7B,C). The average volume of WMH in cerebral cortex of AD group was the highest. For FTLD group, results revealed that the WMH burden in the periventricular region was significantly higher than that in AD (Figures 7D,E). Neither AD group nor FTLD group had subatentorial white matter lesions (Figures 7D,E).

## Discussion

Frontotemporal lobar degeneration (FTLD) and AD are the two main neurodegenerative diseases that cause dementia. Despite recent progress in the early characterization of both disorders, early clinical diagnosis remains a challenge. In this retrospective study, we evaluated the neuropsychological and multimodal neuroimaging properties of the clinical syndromes of AD and FTLD in a Chinese population. We compared biomarker or PET-CT-defined patients and analyzed the first-episode clinical features, the evolution of cognitive function and behavioral and psychological symptoms of these two diseases. Patients with AD often showed cognitive rather than behavioral symptoms at the initial symptoms. As the disease progresses, attention and orientation dysfunction become prominent and gradually accompanied by psychiatric and behavioral symptoms. However, in FTLD group, dysexecutive and language dysfunction features presented as the primarily cognitive phenotypes, accompanied by a certain degree of behavioral abnormalities, and memory deterioration from middle-to-late stage (Figures 3, 4). The prevalence of vascular disease associated factors and APOE ε4 were higher in AD patients than that in FTLD group (Table 1). AD patients were characterized by major atrophy of temporal parietal and relatively sparse of frontal gray matter, whereas FTLD patients typically present with frontal and temporal lobe involvement (Figures 4–6). In addition, we found differences in the burden and distribution of WMH on T2-weighted MRI between AD patients and FTLD patients. The WMH burden of the former was significantly higher than that of the latter, especially in cortical area, while the WMH burden in the periventricular areas of the FTLD patients was higher than that of AD patient (Figure 7), suggesting potentially different underlying neuropathological processes.

### Clinical features and neuropsychological profiles

Some progress has been made in using neuropsychological methods to analyze the differences between FTLD and AD from different manifestations in the cognitive field, but the different stages of the disease are poorly understood. The time sequence of clinical symptoms in the course of disease is the most reliable basis for the correct diagnosis. According to current criteria, memory impairment is not required for the early diagnosis of FTLD, or even as an exclusion criterion of early disease. However, memory loss and visuospatial orientation problems are usually the diagnostic criteria for early symptoms in AD patients, including core clinical symptoms (Gorno-Tempini et al., 2011; Rascovsky et al., 2011; Dubois et al., 2014). By comparison, FTLD patients may develop early neuropsychiatric symptoms, such as agitation and psychosis, consistent with a range of neurological and mental disorders (Rascovsky et al., 2011). Our observational data showed that although all patients with bvFTD met the core clinical symptoms of possible diagnostic criteria at admission, most patients were just close to the threshold of diagnostic criteria at the onset of symptoms [0 feature: 18/106 (16.98%), 1 feature: 23/106 (21.70%) or 2 features: 29/106 (27.35%), Figure 2C], and some patients even have cognitive impairment [20/106 (18.87%), Figure 2B], or accompanied by motor disorders [10/106 (9.43%), Figure 2B] as the onset of symptoms. As a result, these patients are often initially misdiagnosed as psychiatric and other neurological diseases, most commonly AD. Therefore, misconceptions about the early symptoms of both diseases often delay a correct diagnosis.

In this study, through the subitem analysis of MMSE and MoCA, we found that both FTLD and AD patients had memory impairment, but AD patients showed more significant impairment, accompanied by obvious disorientation. However, orientation was relatively preserved in FTLD patients, which was related to the reservation of temporal-parietal lobe combined cortex, anterior cuneate lobe, and posterior cingulate gyrus in early FTLD patients (Figures 4–6). Through the clinical observation of patients with FTLD, it was found that the impairment of executive function was earlier and more severe than memory loss, but the orientation ability and visuospatial ability were preserved to a certain extent (Figures 3A–D). By stratifying the severity of dementia, we found that the degree of cognitive impairment was directly proportional to the severity of dementia in both groups. AD patients almost spread to cognitive areas such as attention, orientation, and language in the middle and later stages of the disease, while FTLD patients mainly showed executive function, attention and language dysfunction, and gradually appeared orientation and memory domains, which was basically consistent with the dynamic manifestations of clinical symptoms (Figures 3A–D).

It is worth noting that, there were remarkable differences between MMSE scores and MoCA scores in FTLD patients; however, the total score of MMSE and MoCA was almost identical in the AD population (Table 1). This is because the MMSE examination focuses more on the detection of memory and visuospatial ability, and these two cognitive areas are the main manifestations of cognitive impairment in AD patients. Therefore, MMSE can accurately reflect the actual degree of cognitive impairment in AD patients. However, due to lack of in-depth monitoring of executive function and language, clinical findings are not very sensitive to FTLD screening. In some patients with FTLD, MMSE is in normal range, but MoCA examination has indicated moderate damage. In the early stage of FTLD, executive ability and language dysfunction are the main cognitive manifestations, and both cognitive areas are reflected in the MoCA subscales, such as Trail Making Test and complex sentence repetition. Freitas et al. (2012) proposed that MoCA had better identification ability than MMSE through comparative study of AD and FTLD. When MoCA score is below 17 points, the sensitivity and specificity for FTLD were 78% and 98%, which were significantly higher than MMSE.

### Potential risk factors

It is suggested that, at the onset of dementia, some clinical and demographic data can be used as predictors of differential diagnosis and future progression. In this study, we have investigated some of the demographic factors most relevant to the cognitive traits, such as age at disease onset, disease duration, and education (Table 1); clinical features as motor signs and behavioral disorders (Figure 2); APOE genotype (Table 1) and vascular diseases and medical histories (Figure 2 and Table 1). In recent years, it has been suggested that AD and FTLD might be kinds of cognitive disorder caused by both vascular pathological changes and neurodegenerative damage, especially in the AD spectrum (Nichols et al., 2021). Many previous longitudinal studies have also shown that vascular risk factors (VRFs) are hazard factors for AD, such as middle-age hypertension, hypercholesteremia, diabetes, obesity, stroke, atrial fibrillation, and lack of exercise (Yu et al., 2020). Our study was consistent with previous reports that the incidence of vascular disease-related histories such as hypertension, diabetes, hyperlipemia, and stroke history seemed over-represented in AD compared to FTLD. Currently, well-controlled VRFs may be one of the reasons for the decline in the prevalence and incidence rate of dementia in some countries (Wu et al., 2017). At the same time, recent researches have shown that VRFs-induced hypoperfusion and hypoxia can lead to amyloid-β accumulation by producing or restraining its degradation, which ultimately impairs neuronal and synaptic plasticity (Pluta et al., 2020; Babusikova et al., 2021). AD patients without VRFs in this study may also have mixed dementia and asymptomatic vasculopathy (Esiri et al., 1999).

Consistent with previous research, the prevalence of APOE ε4 carriers in AD patients (56.84%) was much higher than that in FTLD group (15.09%) in the Chinese cohort. ApoE ε4 has been identified as the strongest genetic predictor of the development of sporadic AD (Ferrari et al., 2018). Furthermore, APOE ε4 carrier status is another key factor of cognitive decline in AD patients with VRFs. APOE ε4 of AD patients are particularly sensitive to VRFs (Lee et al., 2020). Accordingly, APOE ε4 carriage and VRFs may synergistically affect cognitive outcomes in patients with increased genetic and vascular risk (Lee et al., 2020). Functional imaging studies have shown that APOE ε4 carriers not only exhibit substantial reductions in regional cerebral blood flow over time (Thambisetty et al., 2010) but also reduced glucose metabolic rates in posterior cingulate and/or precuneus and lateral parietal lobe (Knopman et al., 2014). Another plausible explanation is that ApoE ε4 allele carriers would disturb the biochemical pathways of neurofibrillary tangle and affect amyloid-β accumulation (Cosentino et al., 2008).

### Neuroimaging distinction between Alzheimer’s disease and frontotemporal lobar degeneration

We identified neuroimaging differences in our study. The current results indicated that symmetry and volume differences in different brain domains, parahippocampal gyrus, entorhinal cortex, and asymmetrical atrophy in putamen and caudate nucleus may distinguish FTLD from AD (Figure 4). Notably, a voxel-based morphometric analysis comparison between the two groups showed a slight effect in the frontal lobe (traditionally supposed to be the core monitor of behavior and executive function) over classical AD, but not as prominent as we observed in FTLD individuals (Figure 4). Although previous cases and our clinical experience have shown that deep prefrontal involvement can be surveyed in patients with frontal variant of AD (Li et al., 2016), these single-subject effects may have been washed in group-level voxel-based morphometric analysis. The discrepancy in frontal atrophy partly explains why psychiatric symptoms, such as hallucinations anxiety and depression are always present in AD patients, but rare in FTLD patients. Complete destruction of frontal lobe function will result in a complete loss of disease awareness in FTLD patients, whereas some of this self-awareness is preserved in AD patients. SBM with non-linearly aligned cortical folding patterns provides precise standardization of participant brains, which may be useful in examining cortical morphology. By measuring cortical thickness, gray to white matter contrast (GWC), surface area, cortical volume, cortical microstructure and macrostructure, SBM provides the possibility to reveal the mechanisms of brain changes and elucidate neurological problems associated with neurodegenerative disease (Singh et al., 2022).

Mapping the distribution and burden of WMH in AD and FTLD will contribute further understand the underlying pathological mechanisms of these diseases. Indeed, previous studies have suggested that the preferential distribution of WMH in cortical regions of AD patients was related to tissue characteristics. For example, as it was located in the watershed areas, normal perfusion in this region was relatively low (Keith et al., 2017). Furthermore, hypersignal of white matter in the paraventricular area is due to periventricular small-vessel disease and neurodegenerative changes, such as amyloid deposition in arteries, arterioles, and veins, leading to brain atrophy and the onset of AD (Keith et al., 2017; Alosco et al., 2018). Conversely, the extensive white matter involvement in cortical and periventricular regions in our FTLD cases has always been without obvious vascular hazard factors or related illness. It has been reported that raised WMH load in symptomatic GRN mutant FTLD patients mainly existed in the frontal and occipital lobes (Sudre et al., 2017).

Although the exact mechanisms of white matter injury in the absence of progranulocyte precursor protein are unclear, it has been hypothesized that the functions of granulocyte precursor protein may play a critical role in neuroinflammation and vascular protection (Ahmed et al., 2007). In addition, other common pathological elements, such as Wallerian degeneration, may be the potential mechanism leading to preferential participation in the distribution of white matter lesions. As a disease with a strong genetic background, we will further analyze the relationship between genotype, neuropathology, neuroimaging, and clinical phenotype, as well as the underlying mechanisms in the subsequent research work.

### Strengths and limitations

To the best of our knowledge, this is the only one of the largest series of direct comparison against well-characterized AD and FTLD patients in a Chinese Han population cohort. The detailed clinical, neuropsychiatric, and multimodal neuroimaging comparisons between these two patient populations would be crucial for future clinical trials. Our rigorous criteria based on detailed clinical examination, neuropsychological, and multimodal neuroimaging with FDG-PET, 3.0T structural MRI, and PiB PET amyloid assays, which enabled us to investigate relationships between neuroanatomical locations of atrophy or WMH, with neuropsychological and neuropsychiatric manifestations, facilitating early diagnosis of neurodegenerative disease. There are also some limitations. **First**, this is a case-selective clinical retrospective study, the recording of clinical data is inevitably incomplete, although we often make a decision with two experienced neurologists, sometimes the subjective judgment of clinicians is inevitable. Therefore, selective bias in clinical data should be taken into account. In addition, our enrolled patients were not racially diverse, therefore, enlarged samples from multi-centers and multi-ethnic are required for comparative analysis of different clinical subtypes. **Second**, the fact that not all patients had PiB PET, thereby the likelihood of mixed clinical superposition cannot be ruled out, because of the clinical and pathological heterogeneity of these two types of neurodegenerative dementia. **Finally**, it is worth mentioning that when we summarized the multimodal imaging features, we did not stratify the different subtypes and different disease stages of the two groups. The heterogeneous composition of populations with different disease stages and baseline levels will partly affect the parameter analysis between clinical characteristics and neuroimaging biomarkers. Since the cross-sectional approach is not conducive to establishing a direct correlation between each clinical scale and neuroimaging changes, longitudinal studies are needed to reflect the direct correlation between the longitudinal performance of each scale, as well as the longitudinal neuroimaging evaluation of MRI parameters during the course of the disease and disease severity.

## Conclusion

We performed a detailed clinical, neuropsychiatric, and multimodal neuroimaging analysis of AD patients compared with FTLD patients. We identified several differences, most importantly, the initial symptoms of the disease and clinical features in the progression of the disease. In addition, medical history, especially vascular disease and associated risk factors, involved brain regions and WMH burden and regional distribution gained from multimodal neuroimaging, may provide valuable supplements for early differential diagnosis. Further studies are warranted to investigate the genetics, neuropathology, biomarkers, and mechanistic pathways to track the course of the disease.

## Data availability statement

The original contributions presented in this study are included in the article/Supplementary material, further inquiries can be directed to the corresponding author.

## Ethics statement

All the subjects were accompanied by reliable caregivers, and the subjects and their families signed the informed consent form. All procedures are carried out according to the ethical standards specified by Tianjin Human trial Committee and approved by Ethics Committee of Tianjin Huanhu Hospital. The patients/participants provided their written informed consent to participate in this study.

## Author contributions

PL conceived and monitored the research. HC, YC, and ZT performed the data collection. PL and WQ analyzed the data, wrote the original manuscript, and made modified. YZ, YW, and MZ participated in clinic diagnosis. YL and HZ were responsible for patient care and scales assessment. All authors examined the results and authorized the final version of the manuscript.

## Footnotes

1
http://fsl.fmrib.ox.ac.uk/fsl/fslwiki/FSL

2
http://stnava.github.io/ANTs/

3
https://cloud.drbrain.net

4
https://neuro-jena.github.io/software.html#vbm

## Supplementary material

Supplementary Figure 1 Sulcus depth, gyrification index and fractal dimension alterations in different groups assessed by surface-based morphometry. **(A)** Comparison between AD patients and healthy controls; **(B)** Comparison between FTLD patients and healthy controls; **(C, D)** FTLD patients compared with AD patients. Color overlay shows *p*_unc_ < 0.001 for family-wise error = 0.05.
