# Supplementary figures and images for: Early-stage differentiation between Alzheimer’s disease and frontotemporal lobe degeneration: Clinical, neuropsychology, and neuroimaging features
Source: Front Aging Neurosci. 2022 Oct 31;14:981451. doi: 10.3389/fnagi.2022.981451 (PMC9659748; doi:10.3389/fnagi.2022.981451)

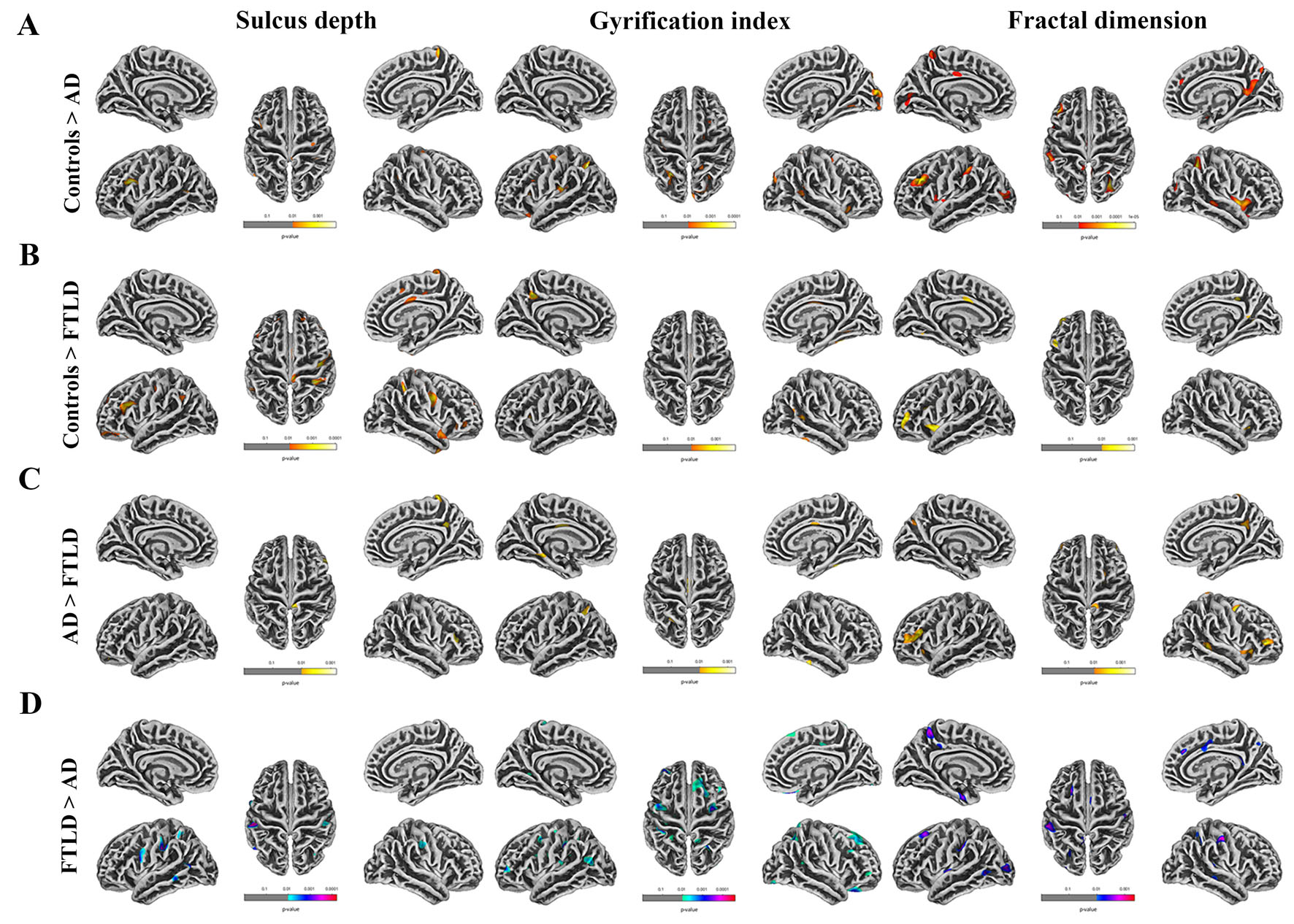

Supplement: Supplementary Figure 1 — Sulcus depth, gyrification index and fractal dimension alterations in different groups assessed by surface-based morphometry. (A) Comparison between AD patients and healthy controls; (B) Comparison between FTLD patients and healthy controls; (C, D) FTLD patients compared with AD patients. Color overlay shows punc < 0.001 for family-wise error = 0.05. [file Image_1.jpeg]
