# Supplementary material for: Early-stage differentiation between Alzheimer’s disease and frontotemporal lobe degeneration: Clinical, neuropsychology, and neuroimaging features
Source: Front Aging Neurosci. 2022 Oct 31;14:981451. doi: 10.3389/fnagi.2022.981451 (PMC9659748; doi:10.3389/fnagi.2022.981451)
Supplement: Supplementary file 2 [file Table_1.DOC]

supplemental table 1. Cortical thickness, sulcus depth, grification index and fractal dimension alterations in patients different groups

| Variables | Brain regions | Peak MNI coordinate  (x, y, z) | Peak intensity  T value (HC-sALS) | *P_FWE-corr_* value |
| --- | --- | --- | --- | --- |
| Controls > AD | | | | |
| Cortical thickness | superior parietal (L) | -45, -24, 46 | 6.48 | 0.000 |
|  | rostral middle frontal (L) | -29, 14, 58 | 6.97 | 0.000 |
|  | lateral occipital (L) | -39, -51, 0 | 3.84 | 0.000 |
|  | fusiform (L) | -34, -45, -5 | 3.30 | 0.000 |
|  | superior temporal (L) | -32, 6, 5 | 3.36 | 0.000 |
|  | inferior parietal (L) | -23, -17, 12 | 3.39 | 0.001 |
|  | superior parietal (R) | 41, -36, 54 | 7.64 | 0.000 |
|  | rostral middle frontal (R) | 27, 31, 16 | 6.30 | 0.000 |
|  | superior temporal (R) | 52, -17, 17 | 3.90 | 0.000 |
|  | superior frontal (R) | 14, 16, 58 | 4.21 | 0.000 |
|  | inferior parietal (R) | 55, -50, 18 | 4.35 | 0.000 |
|  | lingual (R) | 25, -49, 14 | 3.98 | 0.000 |
|  | caudal middle frontal (R) | 32, -3, 64 | 3.90 | 0.000 |
|  | middle temporal (R) | 52, -47, 12 | 3.66 | 0.000 |
|  | lateral occipital (R) | 25, -72, 7 | 4.05 | 0.000 |
| Sulcus depth | pars opercularis (L) | -25, 19, 49 | 3.64 | 0.000 |
|  | inferior parietal (L) | 8, -43, 71 | 2.81 | 0.004 |
|  | paracentral (R) | 8, -43, 71 | 3.17 | 0.001 |
|  | inferior parietal (R) | 56, -47, 38 | 2.80 | 0.004 |
| Gyrification index | superior parietal (L) | -20, -59, 44 | 3.78 | 0.000 |
|  | insula (L) | -24, -14, 31 | 3.87 | 0.000 |
|  | pars opercularis (L) | -23, 25, 36 | 3.88 | 0.000 |
|  | insula (R) | 34, 17, 34 | 3.25 | 0.001 |
|  | superior temporal (R) | 50, 2, 10 | 3.36 | 0.001 |
|  | lingual (R) | 28, -38, 5 | 2.99 | 0.002 |
|  | lateral occipital (R) | 19, -66, -4 | 2.86 | 0.003 |
| Fractal dimension | rostral middle frontal (L) | -33, 25, 52 | 4.22 | 0.000 |
|  | lateral occipital (L) | -23, -71, 6 | 3.15 | 0.002 |
|  | insula (R) | 43, 18, 6 | 3.75 | 0.000 |
|  | inferior parietal (R) | 35, -66, 29 | 4.58 | 0.000 |
|  | superior parietal (R) | 32, -46, 52 | 4.15 | 0.000 |
|  |  | Controls > FTLD |  |  |
| Cortical thickness | postcentral (L) | -32, -13, 43 | 4.87 | 0.000 |
|  | supramarginal (L) | -27, -26, 24 | 5.94 | 0.000 |
|  | caudal middle frontal (L) | -28, 14, 57 | 6.46 | 0.000 |
|  | pars triangularis (L) | -39, 32, 31 | 4.90 | 0.000 |
|  | superior frontal (L) | -4, 7, 58 | 4.54 | 0.000 |
|  | precentral (L) | -39, 12, 36 | 4.13 | 0.000 |
|  | superior temporal (L) | -52, -9, 18 | 4.38 | 0.000 |
|  | precuneus (L) | 4,-52, 30 | 4.08 | 0.000 |
|  | supramarginal (R) | 52, -25, 34 | 6.06 | 0.000 |
|  | precentral (R) | 63, 9, 43 | 5.85 | 0.000 |
|  | superior parietal (R) | 47, -44, 60 | 6.02 | 0.000 |
|  | pericalcarine (R) | 24, -58, 18 | 4.39 | 0.000 |
|  | pars orbitalis (R) | 44, 35, 22 | 4.82 | 0.000 |
|  | rostral middle frontal (R) | 39, 14, 48 | 3.88 | 0.000 |
|  | inferior parietal (R) | 38, -62, 49 | 3.89 | 0.000 |
|  | superior frontal (R) | 17, 6, 56 | 3.70 | 0.001 |
| Sulcus depth | rostral middle frontal (L) | -28, 21, 47 | 3.61 | 0.001 |
|  | lateral orbitofrontal (L) | -7, 62, 19 | 3.24 | 0.002 |
|  | supramarginal (L) | -44, -40,35 | 2.85 | 0.002 |
|  | precentral (R) | 49, -6, 46 | 3.64 | 0.001 |
|  | supramarginal (R) | 39, -38, 42 | 4.13 | 0.000 |
|  | postcentral (R) | 13, -37, 70 | 3.65 | 0.001 |
|  | Superior temporal (R) | 58, 22, 9 | 2.98 | 0.003 |
| Gyrification index | Pars opercularis (L) | -28, 24, 33 | 3.91 | 0.000 |
|  | precuneus (L) | -3, -48, 46 | 3.61 | 0.001 |
|  | lingual (R) | 34, -31, 7 | 3.03 | 0.003 |
|  | inferior temporal (R) | 53, -28, -2 | 3.47 | 0.001 |
| Fractal dimension | insula (L) | -27, 16, 9 | 3.16 | 0.002 |
|  | rostral middle frontal (L) | -21, 61, 30 | 2.96 | 0.004 |
|  | lingual (R) | 31, -47, 16 | 3.28 | 0.002 |
|  |  | AD > FTLD |  |  |
| Cortical thickness | postcentral (L) | -46, -16, 35 | 3.00 | 0.002 |
|  | pars triangularis (L) | -46, 54, 13 | 3.87 | 0.000 |
|  | rostral middle frontal (L) | -36, 76, 28 | 2.84 | 0.004 |
|  | lateral orbitofrontal (R) | 30, 69, -14 | 3.26 | 0.001 |
|  | precentral (R) | 56, 40, 7 | 3.10 | 0.002 |
|  | paracentral (R) | 9, 0, 55 | 2.86 | 0.004 |
| Sulcus depth | lateral orbitofrontal (L) | -19, 92, -6 | 2.53 | 0.008 |
|  | pars triangularis (R) | 47, 63, 14 | 3.26 | 0.001 |
| Gyrification index | superior parietal (L) | -26, -38, 20 | 2.97 | 0.003 |
|  | posterior cingulate (L) | 1, 18, 20 | 3.12 | 0.002 |
|  | inferior temporal (R) | 52, 20, -39 | 3.32 | 0.001 |
| Fractal dimension | rostral middle frontal (L) | -39, 56, 24 | 3.21 | 0.001 |
|  | lateral orbitofrontal (L) | -33, 65, 0 | 2.95 | 0.003 |
|  | superior frontal (R) | 20, 24, 44 | 3.53 | 0.001 |
|  | middle temporal (R) | 43, 1, -20 | 3.18 | 0.001 |
|  | rostral middle frontal (R) | 36, 76, 12 | 3.21 | 0.001 |
| FTLD > AD | | | | |
| Cortical thickness | superior parietal (L) | -38, -25, 14 | 2.78 | 0.004 |
|  | lateral occipital (L) | -46,-18,-28 | 2.53 | 0.008 |
|  | precuneus (L) | -11,-17,17 | 2.80 | 0.004 |
|  | superior parietal (R) | 38, -20, 22 | 3.22 | 0.001 |
| Sulcus depth | supramarginal (L) | -52, -6, 24 | 4.02 | 0.000 |
|  | inferior parietal (L) | -45, -46, -22 | 3.35 | 0.001 |
|  | precentral (L) | -57, 39,16 | 3.30 | 0.001 |
|  | middle temporal (L) | -60, 3, -25 | 3.14 | 0.002 |
|  | superior parietal (R) | -32, -32, 27 | 2.78 | 0.004 |
|  | postcentral (R) | 49, 7, 25 | 2.74 | 0.005 |
|  | inferior parietal (R) | 41, -41, 10 | 2.53 | 0.008 |
| Gyrification index | supramarginal (L) | -35, -5, 16 | 4.31 | 0.000 |
|  | inferior parietal (L) | -49, -27, -18 | 3.13 | 0.002 |
|  | rostral middle frontal (L) | -34, 85, 21 | 3.68 | 0.000 |
|  | pars opercularis (L) | -40, 53, 13 | 3.14 | 0.002 |
|  | superior parietal (L) | -36, -28, 21 | 3.03 | 0.002 |
|  | superior frontal (R) | 20, 41, 41 | 3.06 | 0.002 |
|  | superior parietal (R) | 33, -33, 10 | 3.56 | 0.001 |
|  | lateral orbitofrontal (R) | 12, 73, -17 | 3.74 | 0.000 |
|  | posterior cingulate (R) | 12, 1, 28 | 2.91 | 0.003 |
|  | lateral occipital (R) | 33, -36, -47 | 3.20 | 0.001 |
| Fractal dimension | supramarginal (L) | -51, -7, 20 | 3.25 | 0.001 |
|  | precuneus (L) | -6, -36, 23 | 3.19 | 0.001 |
|  | middle temporal (L) | -43, -27, -29 | 3.28 | 0.001 |
|  | lateral occipital (L) | -29, -51, -23 | 3.13 | 0.002 |
|  | entorhinal (L) | -21, 43, -24 | 3.20 | 0.001 |
|  | insula (L) | -36, 35, -6 | 3.38 | 0.001 |
|  | superior frontal (L) | -23, 31, 54 | 3.13 | 0.002 |
|  | postcentral (R) | 51, 10, 34 | 3.29 | 0.001 |
|  | middle temporal (R) | 42, 17, -21 | 2.64 | 0.006 |
|  | isthmus cingulate (R) | 6, -9, -2 | 2.88 | 0.003 |
|  | superior temporal (R) | 37, 9, -4 | 2.77 | 0.004 |
|  | superior parietal (R) | 36, -32, 20 | 2.55 | 0.008 |
